# Effects of mental health self-efficacy on outcomes of a mobile phone and web intervention for mild-to-moderate depression, anxiety and stress: secondary analysis of a randomised controlled trial

**DOI:** 10.1186/s12888-014-0272-1

**Published:** 2014-09-26

**Authors:** Janine Clarke, Judith Proudfoot, Mary-Rose Birch, Alexis E Whitton, Gordon Parker, Vijaya Manicavasagar, Virginia Harrison, Helen Christensen, Dusan Hadzi-Pavlovic

**Affiliations:** Black Dog Institute, Hospital Road, Randwick, NSW 2013 Australia; School of Psychiatry, UNSW Australia, High Street, Kensington, NSW 2052 Australia

**Keywords:** eHealth, Depression, Anxiety, Psychological stress, Self-efficacy, Mobile health, Intervention studies, Work functioning

## Abstract

**Background:**

Online psychotherapy is clinically effective yet why, how, and for whom the effects are greatest remain largely unknown. In the present study, we examined whether mental health self-efficacy (MHSE), a construct derived from Bandura’s Social Learning Theory (SLT), influenced symptom and functional outcomes of a new mobile phone and web-based psychotherapy intervention for people with mild-to-moderate depression, anxiety and stress.

**Methods:**

STUDY I: Data from 49 people with symptoms of depression, anxiety and/or stress in the mild-to-moderate range were used to examine the reliability and construct validity of a new measure of MHSE, the Mental Health Self-efficacy Scale (MHSES). STUDY II: We conducted a secondary analysis of data from a recently completed randomised controlled trial (*N* = 720) to evaluate whether MHSE effected post-intervention outcomes, as measured by the Depression, Anxiety and Stress Scales (DASS) and Work and Social Adjustment Scale (WSAS), for people with symptoms in the mild-to-moderate range.

**Results:**

STUDY I: The data established that the MHSES comprised a unitary factor, with acceptable internal reliability (Cronbach’s alpha = .89) and construct validity. STUDY II: The intervention group showed significantly greater improvement in MHSE at post-intervention relative to the control conditions (*p*’s < = .000). MHSE mediated the effects of the intervention on anxiety and stress symptoms. Furthermore, people with low pre-treatment MHSE reported the greatest post-intervention gains in depression, anxiety and overall distress. No effects were found for MHSE on work and social functioning.

**Conclusion:**

Mental health self-efficacy influences symptom outcomes of a self-guided mobile phone and web-based psychotherapeutic intervention and may itself be a worthwhile target to increase the effectiveness and efficiency of online treatment programs.

**Trial registration:**

Australian New Zealand Clinical Trials Registry ACTRN12610000625077.

## Background

Online self-management of depression and anxiety has evolved as a popular, clinically effective and cost-efficient public health solution to reducing the personal and societal burden associated with unmet treatment need [1,2]. Grounded predominantly in cognitive behaviour therapy (CBT), and increasingly incorporating other therapeutic approaches [3,4], online psychological interventions help people with symptom management by teaching skills to regain control over and change problematic thoughts and behaviours (including cognitive restructuring, problem solving techniques and behavioural activation [5]). Whereas effect sizes in studies of online interventions compare well with face-to-face treatments [6], the psychological mechanisms that explain these findings are largely unknown. Understanding how, why and for whom interventions affect symptom change is critical for maximising the clinical potency and cost effectiveness of online public health interventions for common mental disorders. Furthermore, rates of adherence with these interventions, which are characteristically low [7], may be improved by incorporating program content and functions that increase therapeutic efficiency by targeting intervening processes directly [8].

A potential framework for understanding the effects of online interventions for mental health problems is provided by Bandura’s Social Learning Theory (SLT; [9]), a theory that specifies multiple interacting determinants of behaviour and behaviour change. According to Bandura, a putative contributor to therapeutic outcomes in psychological interventions is perceived self-efficacy, that is, the degree to which an individual believes that he or she can perform a specific behaviour or set of behaviours. In support of Bandura, self-efficacy has been identified as a key factor explaining treatment gains and behavioural change in several studies of health promoting behaviours, including smoking cessation, reducing alcohol and drug use, weight loss, and chronic disease self-management e.g., [10-14]. Findings show that higher levels of pre-treatment self-efficacy and increased self-efficacy over the course of treatment are important predictors of therapeutic success, and suggest that precise targeting of self-efficacy antecedent processes and information cues may assist in honing treatment efficiency and efficacy [9].

Theoretical models posit that self-efficacy impacts therapeutic outcomes by affecting individuals’ decisions to change their behaviour, and by influencing “*how much effort people will expend and how long they will persist in the face of obstacles and aversive experiences*” ([9] p. 194). Self-efficacy is likely, therefore, to be an important factor contributing to symptom and functional gains within the context of online interventions, particularly those with minimal therapist input. This is because such interventions require the active cognitive and behavioural involvement of the individual [15,16], as well as the ongoing practice and implementation of therapeutic skills (e.g., self-monitoring, activity scheduling, and problem solving; [15,16]), often in the face of challenges and difficult experiences.

Previous reviews support a mediating role of cognitive variables (including dysfunctional attitudes, automatic thoughts and attributional styles) in recovery from mental health problems [17,18] and, more recently, a construct related to self-efficacy, namely ‘perceived control’, has been shown to predict outcomes of online therapist-assisted CBT for depression [4]. Increased self-efficacy beliefs have also been linked with more effective emotion regulation and psychosocial functioning [19]. However, to our knowledge, no research has examined whether symptom and functional outcomes in online self-help interventions are associated with changes in self-efficacy beliefs over the course of treatment (i.e., that improvements in self-efficacy account for treatment gains), and pre-treatment self-efficacy remains largely unexplored as a potential determinant of therapeutic gains in online CBT interventions (that is, self-efficacy as moderator of treatment outcomes).

Randomised controlled trials (RCTs) provide the ideal context in which to examine possible determinants of psychotherapy outcomes [20]. In a recently conducted large scale RCT, we showed that a fully-automated public health intervention combining mobile phone and web technology, *myCompass*, effectively reduced symptoms of depression, anxiety and stress and improved work and social functioning for people with symptoms in the mild-to-moderate range [21]. This paper reports outcomes of a secondary objective of the RCT, namely, to explore the possibility that self-efficacy contributes to symptom improvement and functional gains. Specifically, using data collected at baseline and post-intervention, we tested the hypotheses that: (a) use of the mobile phone and web intervention would increase people’s confidence in their ability to manage their mental health problems, that is their mental health self-efficacy (MHSE), relative to active control (AC) and waitlist (WL) conditions; (b) MHSE would account for all or part of the effect of the intervention on mental health symptom and functional outcomes (i.e., MHSE mediates the treatment effect); and (c) the effect on outcomes of the intervention would differ for those with high and low pre-intervention levels of MHSE (i.e., MHSE moderates the treatment effect).

Self-efficacy is a task-specific construct that varies across distinct groups of behaviours [22]. In contrast with the plethora of self-efficacy scales for physical health and lifestyle improvement, we were able to locate only one scale measuring people’s confidence in managing mental health issues [23]. Developed and validated for use in people with severe mental illness, Carpinello et al.’s [23] Mental Health Confidence Scale relies heavily on recovery-related items, including items referring to mental illness diagnosis and treatment, and may be inappropriate for people with symptoms in the mild-to-moderate range who are unlikely to consider themselves unwell, meet diagnostic criteria or seek treatment [24]. Accordingly, in order to investigate the effects of self-efficacy on therapeutic gains in online psychological interventions, we developed and psychometrically evaluated a new measure of MHSE for common mental health problems, the Mental Health Self-efficacy Scale (MHSES).

## Methods

We report on outcomes of two studies: the development and psychometric evaluation of the MHSES (Study I), and secondary analysis of data from a recently completed RCT to examine the effects of MHSE on symptom and functional outcomes of a fully-automated mobile phone and web intervention (Study II). For both studies, written consent was provided by study participants and ethical approval was obtained from the Human Research Ethics Committee at UNSW Australia (The University of New South Wales; HREC100019). The RCT was registered as Australian New Zealand Clinical Trials Registry ACTRN12610000625077 [https://www.anzctr.org.au/Trial/Registration/TrialReview.aspx?ACTRN=12610000625077].

### Study I

#### Participants and procedure

Participants were 49 people recruited online via the Black Dog Institute’s website and volunteer research register for a proof of concept study assessing the feasibility and acceptability of the myCompass intervention [25]. Eligibility criteria included: having self-reported mild-to-moderate symptoms of depression, anxiety and stress; being an Australian resident aged 18 to 75 years; owning an internet-enabled mobile phone; having access to a desk-top computer with internet capability; and having a valid email address. The initial sample was 70.5% female with a mean age of 38.2 years (*SD* = 12.6).

Participants completed online questionnaires before and after using the myCompass program for six weeks. myCompass is a fully-automated public health CBT intervention for common mental health problems that is delivered via the internet to people’s mobile phones and desk-top computers [21]. Study I reports data collected from participants at baseline.

#### Measures

*The Mental Health Self-efficacy Scale* or MHSES was developed by the authors according to Bandura’s [22] guidelines for constructing self-efficacy questionnaires. An initial pool of items derived from Bandura’s theory of self-efficacy and assessing belief in one’s capability to perform behaviours related to mental health self-care was reduced by agreement among the investigators to six items, with each presented as a question (e.g., “*How confident are you that you can make your days moderately enjoyable*?”). Participants rated each statement on a 10-point Likert scale ranging from 1 (“*Not at all confident*”) to 10 (“*Totally confident*”). Table 1 contains the six MHSES items.

**Table 1:** **The Mental Health Self-efficacy Scale (MHSES): Items and results of exploratory FA**

**MHSES items**	**Factor loadings**	**Communalities**	**Cronbach’s alpha if item deleted**
Please read each question and rate how **confident** you are that, on an average day in the next month, you will be able to do the following things.			
**On an average day in the next month, how confident are you that…**			
1. You can keep your stress, anxiety or depression from interfering with the things that you want to do?	.75	.56	.88
2. You can do the different tasks and activities needed to manage your stress, anxiety or depression so as to reduce your need to see a doctor?	.89	.80	.86
3. You can do things other than just taking medicine to reduce how much your stress, anxiety or depression affects your everyday life?	.73	.53	.88
4. You can make your days at least moderately enjoyable?	.74	.54	.87
5. You will have moderate amounts of time where you do not experience stress, anxiety or depression?	.61	.37	.89
6. You will be able to effectively manage any stress, anxiety or depression that you do experience?	.85	.72	.86

*The Depression, Anxiety and Stress Scales* or DASS [26] is a widely used self-report measure of depression, anxiety and stress. The DASS has high internal consistency, acceptable test-retest reliability [26] and yields reliable and valid data when used in an online format [27]. Respondents are asked to indicate the frequency with which they experienced symptoms of depression, anxiety and stress over the previous week. Total scores range from 0 to 126 and subscale scores range from 0 to 42, with higher scores indicating greater symptom severity.

*The Work and Social Adjustment Scale* or WSAS [28] assesses the degree to which mental health problems interfere with day-to-day functioning in five domains: work, social leisure activities, private leisure activities, home-management, and personal relationships. It provides an assessment of the experiential impact of mental health symptoms from the sufferer’s point of view, with higher scores indicating poorer adjustment (range 0 to 40). Meyer et al. [5] provide data supporting the psychometric adequacy of the WSAS when administered in an online format.

*The 10-item Personality Inventory* or TIPI [29] was administered at baseline only. The TIPI contains five two-item subscales measuring the five personality factors (openness to experience, conscientiousness, extraversion, agreeableness and neuroticism). Scores on each subscale range from 2 to 14, with higher scores indicating higher levels of each trait.

#### Analyses

Statistical analyses were completed with SPSS 21.0 software. Descriptive statistics were calculated for the DASS, WSAS and TIPI. Recommendations for the optimal subjects-to-variables ratio in factor analytic studies vary considerably. As our sample exceeded the widely accepted ratio of at least five subjects per variable [30], maximum likelihood factor analysis (FA) with varimax rotation was used to examine the dimensionality of the MHSES and determine the final composition of the Scale. Internal consistency reliability of the MHSES was assessed using Cronbach’s alpha, and construct validity was determined using Pearson’s correlation to relate baseline scores on the MHSES to theoretically related constructs [9,15,19], including baseline measures of symptoms and overall psychological distress (DASS), work and social adjustment (WSAS) and neuroticism, an aspect of personality that comprises a lack of emotional stability and confidence (assessed by the TIPI).

### Study II

#### Participants and procedures

A detailed description of the study participants and procedures is provided in Proudfoot et al. [21]. Seven hundred and twenty people, recruited via the internet, radio and print media advertising and meeting the same criteria as for Study I, participated in Study II. The sample was predominantly female (*n* = 491, 69.6%), university educated (*n* = 387, 53.7%), employed (*n* = 591, 83.8%) and married (*n* = 288, 41%), with a mean age of 38.9 years. Participants were randomised after baseline to one of three conditions: myCompass (*n* = 242), an attention control condition (*n* = 248) and a waiting list control group (*n* = 230).

#### Interventions

Participants in the myCompass condition were able to use the program, *ad libitum,* for seven weeks. Attention control participants received a control mental health program with high face validity that was matched to the myCompass intervention on duration and mode of delivery. The program was designed to be interesting but contained no management advice or therapeutic strategies. Following a 7-week delay, waitlist participants received full access to the myCompass program for seven weeks.

#### Measures

All of the measures completed in Study I, with the exception of the TIPI, were completed online by participants in Study II at baseline and post-intervention (eight weeks).

#### Analyses

Statistical analyses were completed with SPSS 21 software. In the first instance, we used baseline data to re-examine the psychometric properties of the MHSES using similar procedures to those conducted in Study I. However, on the basis of the results of the exploratory FA, confirmatory factor analysis (CFA) was conducted for the MHSES to further study its construct validity.

According to Baron and Kenny [31], MHSE would satisfy criteria for mediation if: (1) symptom improvement and functional gains were greatest for participants who used the mobile phone and web intervention; (2) change in MHSE was greatest for participants in the intervention condition; (3) change in MHSE was associated with change in symptoms and work and social functioning; and (4) the effect of the intervention on symptom and functional outcomes was attenuated after controlling for the direct effect of MHSE. Although the necessity for mediation of a direct effect on outcomes has since been questioned (criterion 3 [20,32]), Baron and Kenny’s [31] causal steps framework is still the most widely used approach to testing mediation in the social sciences [33].

Statistical methods for testing mediation vary, so we examined our data using two techniques. Initially, Baron and Kenny’s criteria were examined sequentially in a series of mixed models repeated measures (MMRM) procedures. In MMRM, no participant is removed from the analysis because all available data are used to obtain parameter estimates. In the present study, restricted maximum likelihood (REML) was used to estimate model parameters, and error degrees of freedom were calculated using Satterthwaite’s approximation [34]. Analyses assumed a compound symmetric structure, in line with Fairclough’s recommendation that the covariance structure be restricted in situations where attrition is high [35].

In a second set of analyses, direct and indirect effects of MHSE on outcomes were examined using Preacher and Hayes’ [32] revised version of the Sobel test [36]. Their revision uses bootstrap samples to compute parameter estimates for direct and indirect effects and bias corrected 95% confidence intervals, and does not require the sampling distribution of indirect effects to be normal. For this reason, the procedure is increasing in popularity as a statistically rigorous approach for assessing mediation in treatment outcome studies [37,38].

In both sets of analyses, MHSE reflected change from baseline to post-intervention, and was computed by subtracting baseline scores from post-intervention scores [38]. Baseline scores on the outcome variable of interest were entered as covariates in all models.

To explore the potential moderating effect of MHSE, an additional set of MMRM procedures was conducted, which included all possible main effects and interactions between treatment condition, time, and baseline MHSE. Again, baseline scores on the respective outcome measures were entered as covariates in the analyses.

## Results

### Study I

An initial FA of the six MHSES items yielded a single factor solution accounting for 67% of the cumulative variance. As shown in Table 1, factor loadings for items ranged between 0.61 and 0.89, and were substantially higher than the 0.3 to 0.4 criterion that is commonly used in factor interpretation and questionnaire design [39,40]. The communality for one item (item 5) was marginally lower than the generally accepted minimum criterion of 0.40 [30], however, its deletion did not improve Cronbach’s alpha (0.89) so a decision was made to retain this item.

Descriptive statistics for the DASS subscales, the WSAS, the MHSES and the TIPI are presented in Table 2. For each participant, ratings across the six MHSES items were summed to obtain an overall measure of their MHSE, with higher scores indicating greater self-efficacy (scores range from 10 to 60; see Table 2). Total scores on the MHSES correlated significantly and negatively with DASS Depression (*r* = −0.41, *p* = .005), DASS Total (*r* = −0.31, *p* = 0.048) and WSAS (*r* = −0.48, *p* = 0.001) scores. Whereas higher MHSE was associated with greater emotional stability on the TIPI (*r* = 0.40, *p* = 0.007), no other correlation between the MHSES and TIPI subscales achieved significance.

**Table 2:** **Means (standard deviations) and correlations with the Mental Health Self-efficacy Scale (MHSES) for baseline measures**

**Measure**	**Mean (SD)**	**Correlation with MHSES**
DASS (n = 44)		
Depression	16.55 (9.90)	-.41**
Anxiety	8.95 (8.25)	-.23
Stress	19.23 (8.22)	-.08
Total Score	44.72 (21.66)	-.31*
WSAS	23.48 (7.66)	-.48**
TIPI		
Extraversion	6.48 (3.20)	.17
Agreeableness	10.05 (2.46)	.11
Conscientiousness	9.73 (2.61)	.13
Emotional stability	6.09 (2.78)	.40**
Openness	9.86 (2.89)	-.06
MHSES	33.23 11.45	-

*p < 0.05, **p < 0.01.

### Study II

#### Psychometric properties of the MHSES

A CFA testing the validity of the factor structure derived for the MHSES in Study I yielded a chi-square of 146.7 (*df* = 9, *p <* = 0.001), and the following fit indices: comparative fit index (CFI) = 0.95; Tucker-Louis Index (TLI) = 0.91; root mean square error of approximation (RMSEA) = 0.15; and standardised root mean square residual (SRMR) = 0.03. Whereas the CFI, TLI and SRMR all satisfied conventional guidelines for acceptable model fit (i.e., CFI and TLI > 0.90, and SRMR < 0.08), the RMSEA was larger than the desirable upper limit of 0.08 [41-43]. In light of recent evidence questioning the validity of the RMSEA in models with low degrees of freedom [Kenny DA, Kanisken B, McCoach D: The performance of RMSEA in models with small degrees of freedom. Unpublished paper: University of Conneticut], and given further evidence of the model’s adequacy in the form of significant parameter estimates across all scale items (range 0.75 to 0.85; all *p*s < = 0.001), we judged that there was sufficient evidence confirming the single factor structure of the MHSES. Cronbach’s alpha for the MHSES was 0.91.

Significant correlations in the expected direction between the MHSES and DASS Depression (*r* = −0.53, p < = 0.001), Anxiety (*r* = −0.31, *p* < = 0 .001) and Stress (*r* = −.35, *p <* = 0.001) subscales, DASS Total scores (*r* = −0.51, *p <* = 0.001) and the WSAS (*r* = −0.49, *p* < = 0.001) provided further construct validity for the scale.

#### Mediation analyses

Findings of the MMRM procedures are presented first. We have previously provided support for Baron and Kenny’s [31] first criterion, with data showing that symptom improvement and functional gains were greatest for people who used the myCompass intervention [21].

In the present study, a 3 (groups) by 2 (time) repeated measures model, with the between-subjects variable of group (myCompass, AC and WL) and the within-subjects variable of time (pre-intervention and post-intervention), yielded a significant interaction effect of treatment and measurement occasion for scores on the MHSES [*F*(2, 757.26) = 15.18, *p <* = 0.001]. A set of Bonferroni adjusted interaction contrasts constructed to estimate between group differences in mean change from baseline to post-intervention showed significantly greater improvement in MHSE for participants in the mobile phone and web intervention condition than the AC (*p* = 0.000) and WL (*p* = 0.000) groups. These data provided support for criterion 2.

Criterion 3 and 4 were tested simultaneously in MMRM analyses that included the effect of change in MHSE on symptom and functional outcomes. Table 3 summarises the results of these analyses and shows support for a mediating effect of MHSE on anxiety and stress outcomes.

**Table 3:** **Tests of group x time interaction after controlling for the effect of mental health self-efficacy on symptoms and functional outcomes**

	**Group by time**			**Mental health self-efficacy**		
**Outcome**	***df*** **(numerator, denominator)**	***F***	***p***	***df*** **(numerator, denominator)**	***F***	***p***
DASS Depression	2,472.60	9.21	.000	1,493.18	34.70	.000
DASS Anxiety	2,474.23	2.69	.070	1,489.23	9.96	.002
DASS Stress	2,473.33	2.27	.104	1,493.02	16.99	.000
DASS Total	2,473.15	6.17	.002	1,493.14	31.56	.000
Work and social functioning	2,474.45	5.40	.005	1,491.14	13.78	.000

Table 4 presents the results of the bootstrapping analyses. In this case, support for mediation is demonstrated by a non-significant *c*’ path (direct effect of treatment on outcomes) in the presence of significant *a* (effect of treatment on mediator), *b* (effect of mediator on outcome), and *c* (total effect of treatment on outcome) paths. Findings replicated the results of the MMRM analyses, providing support for MHSE as a mediator of anxiety and stress outcomes. Interpreting the coefficients for the *b* path, improvements in anxiety and stress symptoms at post-intervention were accounted for by increased MHSE.

**Table 4:** **Results of mediation analyses with bootstrap indirect results**

		**Direct and total effects coefficients**				**Bootstrap indirect effect 95%** **bias corrected CI** ^**†**^	
**Outcome**	**Adjusted** ***R*** ^**2**^	***a***	***b***	***c***	***c’***	**Lower limit**	**Upper limit**
DASS							
Depression	.46	−1.35*	−0.35**	2.10**	1.67**	0.089	0.935
Anxiety	.43	−1.46*	−0.19**	0.81*	0.53	0.063	0.571
Stress	.35	−1.43*	−0.22**	0.98*	0.67	0.067	0.628
Total	.45	−1.45*	−0.77**	3.88**	2.76*	0.256	2.163
WSAS	.49	−1.38*	−0.23*	1.46*	1.14*	0.060	0.637

*p < 0.05, **p < 0.001.

^†^Lower and upper limits of confidence intervals for test of mediation with 5,000 bootstrap resamples and bias correction.

#### Moderation analyses

**Figure 1:**
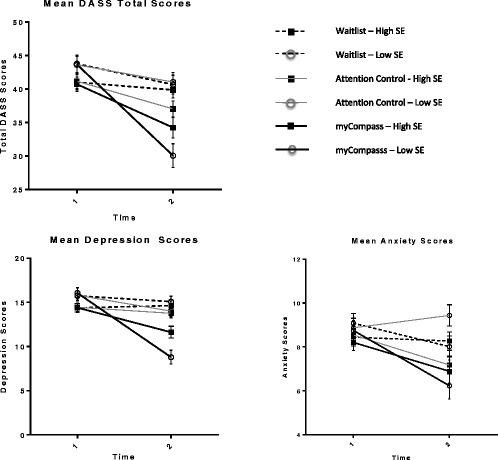
**Moderating effects of mental health self-efficacy on treatment outcomes.**

Results of the MMRM procedures testing moderation are summarised in Table 5. Including a three-way interaction term (Group by Time by Baseline MHSE) as a continuous variable in the analyses revealed a significant moderating effect of MHSE on treatment outcomes for DASS Anxiety, DASS Depression and DASS Total scores. To explore these effects further, we used the median split method to dichotomise baseline MHSES scores and plotted the estimated marginal means for high and low scorers in each condition (See Figure 1). Interaction contrasts comparing the differential effects of high and low MHSE in the intervention condition showed that the mobile phone and web program was most effective in people with low MHSE at baseline (all *p*’s < = .002).

**Table 5:** **Tests of the group x time x mental health self-efficacy (MHSE) interaction on symptom and functional outcomes**

	**Group by time by MHSE (baseline)**		
**Outcome**	***df*** **(numerator, denominator)**	***F***	***p***
DASS Depression	2,716.02	5.60	.004
DASS Anxiety	2716.40	3.68	.026
DASS Stress	2,714.12	1.75	.175
DASS Total	2,715.55	4.40	.013
Work and social functioning	2,717.33	0.83	.431

## Discussion

In the present study, we provide preliminary data on a new scale measuring people’s confidence in managing issues related to their mental health, the MHSES. We also explored hypotheses derived from Bandura’s SLT: first, that symptom and functional gains in a mobile phone and web psychotherapeutic intervention would be mediated by MHSE; and second, that program outcomes would differ between people with high and low levels of pre-intervention MHSE.

Data from both Studies I and II provide support for the MHSES as a parsimonious and reliable measure of MHSE, with high construct validity. Factor analysis showed that the Scale is best considered unidimensional - the high internal consistency estimate providing further evidence that scale items function well together to consistently measure MHSE. Moderate correlations in the expected direction with measures of depressive symptoms, overall psychological distress, work and social functioning and emotional stability support the construct validity of the MHSES, while at the same time indicating that the scale measures a discrete construct. Harrison et al. [25] have previously reported sensitivity of MHSES scores to change, a finding consistent with Bandura’s [9] proposition that self-efficacy is a malleable psychological state, as opposed to a more permanent personality trait. Together, the available data provide preliminary endorsement for the MHSES as a psychometrically sound and easily administered measure of MHSE. Further testing of the measure in other mental health interventions, including face-to-face therapies, is essential, as is comparing the Scale’s results with those derived from measures of other related psychological states, such as generalised self-efficacy, coping skills and perceived control.

In Study II, use of the mobile phone and web-based intervention was associated with increased MHSE, and MHSE was linked with reduced depression, anxiety and stress symptoms, and improved work and social functioning. Importantly, we also found evidence for a potential mediating effect of MHSE on anxiety and stress symptoms, with improvements in MHSE associated with the greatest symptom gains. Together, these findings are in line with studies showing the benefits for health behaviours and physical health outcomes of interventions that enhance self-efficacy [10-14], and are consistent with findings supporting the role of cognitive factors, including perceived control, as mediators of outcomes of face-to-face [17,18] and web-based therapies [4].

Data also identified MHSE as a potential moderator of treatment outcomes in the mobile phone and web-based intervention. Interestingly, while Bandura’s SLT posits greater therapeutic gain for people with high pre-treatment MHSE (due to their perception of tasks as being within their control, as well as their greater motivation, and more active task engagement), we found that users of the intervention with low MHSE typically reported the greatest symptom improvement. One possibility is that gains were greatest for low self-efficacy users because their higher symptom scores at baseline left them with greater potential for improvement. Alternatively, given that individuals with low self-efficacy typically lack confidence and require more guidance in managing activities [44], a self-efficacy enhancing web-based intervention (like myCompass) may provide exactly what they need; the skills, motivation, and self-assurance necessary to better manage their mental health symptoms.

Although unexpected, the finding that MHSE did not mediate or moderate work and social functioning outcomes is most likely reflective of the behaviour-specific nature of SE beliefs [22]. We speculate that MHSE beliefs may be more predictive of people’s functioning in the mental health domain (for example, treatment attendance, medication adherence, and active self-monitoring). This question needs to be explored in further research.

### Implications for program design and clinical practice

The finding that MHSE enhancement mediated symptom improvement suggests that precise targeting of MHSE may have the potential to increase the therapeutic potency and clinical efficiency of online interventions for common mental health problems. Research has shown that self-efficacy can be reinforced via a range of information sources, including performance mastery, verbal persuasion and social influence, vicarious learning, and emotional arousal [15], and studies show that self-management programs incorporating these strategies produce more favourable physical health outcomes [45,46]. In the case of myCompass, Bandura’s SLT may provide a useful theoretical basis upon which the program’s self-efficacy promoting content and functions can be enhanced.

The analyses also indicated a sub-set of individuals with symptoms in the mild-to-moderate range who may indeed benefit most from web-based psychotherapeutic interventions, namely those with low MHSE. Primary care of people with symptoms in this range is often complicated by the fact that providers, especially general practitioners (GP), face difficulties identifying which of their patients will take-up and benefit from the various treatment options available (e.g., face-to-face or online psychotherapy, supportive counselling, and medication; [47,48]). At minimum, our findings suggest that screening of patients using a short, simple, measure of MHSE (such as the MHSES) might be useful for recognising patients who are most likely to benefit from self-help interventions delivered online.

### Study limitations and future research

Some limitations of the study should be noted. First, data were derived from volunteers with mild-to-moderate symptoms who agreed to use a mobile phone and web-based self-help psychotherapeutic intervention. It is possible, therefore, that our findings are not generalisable to non-volunteers, whose decision not to use a self-help intervention may variously reflect people’s low or high confidence that they can self-manage their mental health symptoms. Future studies might shed light on this issue.

Second, although our research design enables us to examine the status of MHSE as a potential mediator of symptom and functional outcomes in web-based interventions [20], we are prohibited from making firm statements about the causal role of the construct in determining treatment gains. It is not possible, for example, for us to discount the possibility that change in MHSE is an epiphenomenon of improved mental wellbeing. A more statistically robust test of mediation would require demonstration of change in MHSE prior to change in symptom and functional outcomes. Alternatively, if another RCT demonstrated increased effectiveness of a web-based psychotherapeutic intervention after more precise targeting of MHSE, then confidence in the causal role of MHSE would increase [20].

Finally, MHSE was the only potential mediator considered in this study, thereby precluding us from commenting on its relative utility in predicting symptom and functional outcomes in web-based psychotherapies. For example, there are other variables from Bandura’s SLT, including outcome expectancies and personal goals [9], that may combine with such cognitive variables as attitudes, thoughts, and attributional styles, to affect outcomes of face-to-face and online therapies. Multiple mediator models in which MHSE is pitted along-side other theoretically relevant mediator variables should be studied. As suggested by our data, it is likely that differences exist in the putative mediators of mental health symptom versus functional outcomes in web-based interventions, with important implications for designing program content and functions.

## Conclusion

The potential role of perceived self-efficacy in determining outcomes of mental health interventions is testable now that a simple, reliable and valid measure of MHSE is available. In Study II, we showed that MHSE is a potential psychological mechanism through which a fully automated, mobile phone and web psychotherapeutic intervention affects symptom and functional outcomes. It also appears that differences in pre-treatment levels of MHSE may have important implications for understanding differential responses to treatment. Together, these findings suggest that perceived MHSE may be an important factor in overcoming mild-to-moderate mental health problems, as well as a worthy and measurable target of program development and research investigating the efficacy and effectiveness of public mental health interventions.
